# Applying Dialysis Bags to Grow Microalgae and Measure Grazing Rates by Secondary Producers

**DOI:** 10.3389/fphys.2022.838001

**Published:** 2022-05-10

**Authors:** Yang Tian, Xiangqi Yi, Kunshan Gao

**Affiliations:** ^1^ State Key Laboratory of Marine Environmental Science, College of Ocean and Earth Sciences, Xiamen University, Xiamen, China; ^2^ Co-Innovation Center of Jiangsu Marine Bio-Industry Technology, Jiangsu Ocean University, Lianyungang, China

**Keywords:** grazing rate, phytoplankton, zooplankton, secondary producer, dialysis bag

## Abstract

Traditional methods using sealed bottles to determine the grazing rates by secondary producers neglect chemical changes induced by biological activities during the incubation, giving rise to instable levels of nutrients, pH, *p*CO_2_, *p*O_2_ and other chemicals along with changing microalgal cell concentrations and grazers’ metabolism. Here, we used dialysis bags, which allows exchanges of nutrients and gases, to grow microalgae and to determine grazing rates of secondary producers. The specific growth rate of diatom within the dialysis bags increased with increasing water velocities, indicating its suitability to grow microalgae under dynamic water conditions. Then, we compared the grazing rates by the heterotrophic dinoflagellate *Noctiluca scintillans* measured with the traditional method using polycarbonate (PC) bottles and the approach with the dialysis bags, and found that these two methods gave rise to comparable grazing rates. Nevertheless, the concentrations of inorganic nitrogen and phosphate in the closed PC bottles were about 89–94% lower than those in the dialysis bags due to the microalga’s assimilation. Subsequently, we applied it to determine the grazing rates by a copepod and an oyster (in the presence of other grazers). Consistent results were obtained using the dialysis bags to determine grazing rates by copepods. During the mesocosm (3000 L) experiment in the presence of primary and secondary producers, the grazing rates by the oyster *Crassostrea angulata* were determined based on the difference of phytoplankton biomass within and outside of the dialysis bags that held all organisms in the mesocosm except the oyster. Since the dialysis bags are permeable to gases, the grazing rates by the oyster under 410 (AC) and 1,000 (HC) μatm CO_2_ were successfully measured, with a promising result that HC significantly increased the oyster’s grazing. We concluded that using dialysis bags to grow microalgae and to determine grazing rates is a reliable approach, especially under different levels of CO_2_ and O_2_.

## 1 Introduction

In aquatic food webs, secondary producers bridge primary producers and higher trophic levels *via* grazing. Therefore, grazing rates are frequently estimated. It can be assessed in different ways, such as dilution method (Landry and Hassett, 1982), intestinal pigment analysis (Mackas and Bohrer, 1976), radioisotope or fluorescence labeling technique (Lessard and Swift, 1985; Sherr and Sherr, 1987) and prey biomass subtraction method (Frost, 1972). Prey biomass subtraction method has been frequently used in laboratory (Ger et al., 2016), and dilution method has been often employed during field studies (Worden and Binder, 2003). However, due to the lack of exchanges of chemicals and gases with milieu, these traditional methods with small closed containers may cause bottle effects (Gao et al., 2012; Gao 2021). The neglected removals of nutrients and changes of aquatic chemistry, therefore, most likely lead to inaccurate assessment of the grazing rates.

Since dialysis membranes are permeable to ions and small molecules, preventing penetrations of larger particles or microbes, dialysis bags have been employed in marine science studies examining the growth rate of single species or phytoplankton community under *in situ* environmental conditions (Jensen et al., 1972; Sakshaug, 1977; Furnas, 1982; Furnas, 1990; Mura and Agusti, 1996), the grazing rates of microzooplankton combined with dilution techniques (Landry and Hassett, 1982; Stibor et al., 2006), viral lysis and protistan predation (Weinbauer et al., 2007), allelopathy (Ma et al., 2015), microalgae-bacteria interaction (Zhang et al., 2019) and deep-sea microbes *in-situ* incubation (Zheng et al., 2021). Apparently, the deficiency in using a completely-closed testing system for grazing measurements could be improved by using the dialysis bags, since nutrients and gases can be easily exchanged between the inner and outside of the bags.

Diatoms and dinoflagellates are two typical phytoplankton groups, playing essential roles in the primary production and biogeochemical cycles. On the other hand, zooplankton and other secondary producers drive energy transfer in trophic webs and contribute to sustainability of marine ecosystems. Therefore, accurate and comparable assessments of growth rates of microalgae and grazing rates of secondary producers are essential in understanding the energy flow and their responses to environmental changes. Here, we used dialysis bags to measure microalgae growth, grazing rates of heterotrophic dinoflagellates and copepods in microcosm and then we applied the dialysis bag approach to measure the grazing rates of oysters in mesocosms. Our results indicate that this approach using dialysis bags can be applied to measure microalgal growth and grazing rates of secondary producers under *in situ* conditions or in mesocosm studies.

## 2 Application of Dialysis Bags for Microalgae Culture and Grazing Rate Measurement

### 2.1 Characterization of Dialysis Membranes

The regenerated cellulose dialysis membranes (Biosharp, Beijing, China) with different molecular weight cut-offs (MWCOs), 7,000 Da (7 KD) and 14,000 Da (14 KD), were cut into sections of 17 cm in length. Since the dry membranes contain glycerin as a humectant and low levels of sulfide and metal ions, they were cleaned and activated according to the manufacturer’s instructions. After being boiled in 2% sodium hydrogen carbonate and 1 mM ethylenediaminetetraacetic acid disodium salt solution (pH 8.0) for 10 min, the membranes were flushed thoroughly by autoclaved reverse osmotic (RO) water and then boiled in 1 mM ethylenediaminetetraacetic acid disodium salt solution (pH 8.0) for another 10 min. The dialysis bags were rinsed further with autoclaved RO water and kept in sterile RO water at 4°C for later uses.

### 2.2 Developing the Relationship of Diffusion Parameters With Water Velocities

In order to quantify the diffusion performance of the dialysis bags with different MWCOs under different water current speeds, we monitored the changes of CuSO_4_ concentration in two compartments (within and outside of the bags), which were separated by the dialysis membrane. The reason for choosing CuSO_4_ as the indicator is that the concentration of copper ions in aqueous solution can be readily measured by a spectrophotometer. Furthermore, nutrients exist generally as ions, and thus the diffusion of Cu^2+^ through the membrane could be considered as a proxy of nutrients (effective hydrated ionic radii are similar among Cu^2+^, nitrate, nitrite, phosphate and ammonium, ranging from 0.25 to 0.6 nm) (Speight, 2005). The standard curve for CuSO_4_ concentration vs. OD_800_ values was constructed using a UV-VIS spectrophotometer (TU-1810, Persee, China). The CuSO_4_ solution of 0.1 mol L^−1^ was injected into each dialysis bag (44 mm in flat width, 17 cm in length, 50 ml). Both ends of the dialysis bag were sealed with polyamide clamps. Then, the sealed bags were placed and fixed in a glass beaker (198 mm in height, 99 mm in diameter) containing 1 L RO water. Different hydrodynamic conditions were obtained by adjusting the speed of a cylindrical polytetrafluoroethylene stirring bar (Ф7 mm, 25 mm in length) driven by a magnetic stirrer (LS-DMS-ProNI, Lichen, China). The velocities at different locations were measured by a portable current meter (LS300-A, Xiangruide, China). The current speeds at the site where the dialysis bags were placed were measured for more than 2 min to gain a stable value. We selected four velocities (0, 1.8, 3.9, 5.1 cm s^−1^) and two types of dialysis bags (7 KD and 14 KD), and each combination has two replicates. The concentrations of CuSO_4_ inside and outside of the bags were measured at 0, 1, 3, 5, 7, 9, 11 and 23 h.

Theoretically, the Cu^2+^ diffusion across the dialysis membranes should follow the Fick’s law of diffusion:

$$\begin{array}{c} J=\;-D\;\left(C_{in}-\;C_{out}\right), \end{array}$$ (1)

where J is the diffusion flux whose dimension is the amount of Cu^2+^ per unit area per unit time, (C_in_–C_out_) is the concentration difference across the dialysis membrane, and D is the diffusion coefficient. With assumed homogeneity of the solution, the corresponding concentrations of Cu^2+^ can be described as:

$$\begin{array}{c} \frac{dC_{in}}{dt}=\;\frac{-\;D\;\;\left(C_{in}-\;C_{out}\right)\;S}{V_{in}}, \end{array}$$ (2)

$$\begin{array}{c} \frac{dC_{out}}{dt}=\;\;\frac{\;D\;\left(C_{in}-\;C_{out}\right)\;S\;}{V_{out}}, \end{array}$$ (3)

where S is the membrane area, V_in_ is the volume of the dialysis bags, and V_out_ is the volume of the beaker. Because the size of the dialysis bags used in this experiment is fixed, we multiplied D and S as a new parameter, apparent diffusion coefficient (D_apparent_). Therefore, the effect of water velocity on the diffusion can be reflected by the D_apparent_. The above differential equations can be solved as follows:

$$\begin{array}{c} C_{in,t}=\;\frac{C_{in,0\;}V_{in}+C_{out,0}V_{out}+\left(C_{in,0}-C_{out,0}\right)e^{\frac{-D_{apparent}\;\left(V_{in}+V_{out}\right)t}{V_{in}\;V_{out}}}V_{out}\;}{V_{in}+V_{out}}, \end{array}$$ (4)

$$\begin{array}{c} C_{out,t}=\;\frac{C_{in,0\;}V_{in}+C_{out,0}V_{out}-\;\left(C_{in,0}-C_{out,0}\right)e^{\frac{-D_{apparent}\;\left(V_{in}+V_{out}\right)t}{V_{in}\;V_{out}}}V_{in}\;}{V_{in}+V_{out}}, \end{array}$$ (5)

where *C*_*in,t*_ is the concentration of the CuSO_4_ within the dialysis bags at time *t*, *C*_*out,t*_ is the concentration of the solute in the beaker at time *t*, *V*_*in*_ is the volume of the dialysis bags, *V*_*out*_ is the volume of the beaker. Based on the measured Cu^2+^ concentrations at different time points, the apparent diffusion coefficient can be obtained through nonlinear regression fitting.

**FIGURE 1:**
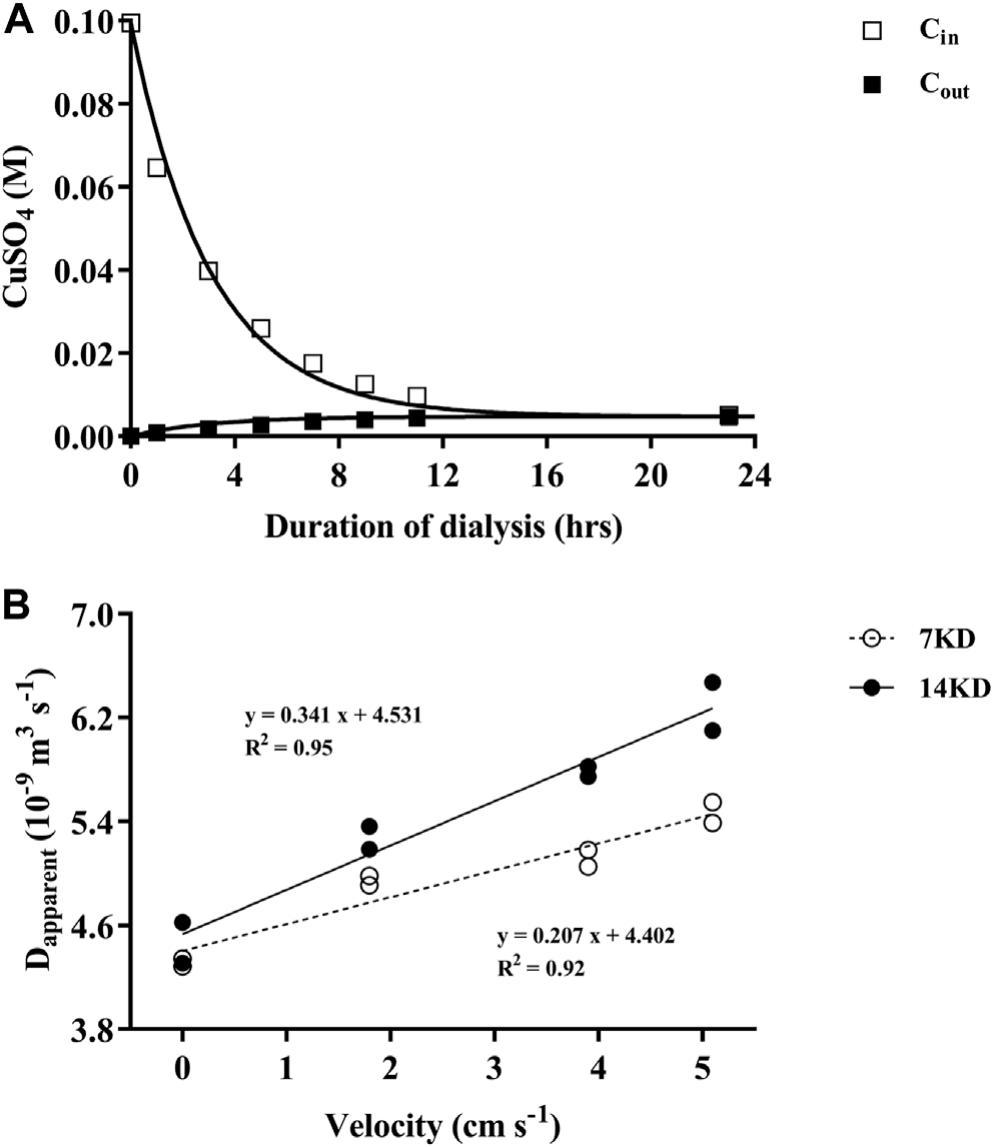
**(A)** The changes of CuSO_4_ concentrations within (C_in_) and outside (C_out_) of the dialysis bags over time, with the curves representing the predicted and the squares the measured values. **(B)** Relationship of the apparent diffusion coefficient (D_apparent_) of the dialysis membranes (7, 14 KD) with water velocities outside of the bags.

The validity of this model we used was confirmed by the effective match between the measured values and the predicted values by the model (Figure 1A). The apparent diffusion coefficient of the dialysis membranes increased linearly with the increasing of the water velocity, and the difference between the two membranes were enlarged with increased water velocities in the beaker (Figure 1B). Due to the relatively higher diffusion performance under water velocities, dialysis bags possessing 14 KD MWCOs were selected for the following experiments.

### 2.3 Testing the Feasibility to Use Dialysis Bags for Microalgal Culture

The centric diatom *Thalassiosira weissflogii* (strain CCMA 102), obtained from the Center for Collections of Marine Algae (Xiamen University, China), was grown at 20°C in f/2 medium under 230 μmol photons m^−2^ s^−1^ (PAR) and a 12:12 h light-dark cycle (Guillard and Ryther, 1962; Guillard, 1975). The microalgal cells in mid-exponential phase were collected by filtration with less than 0.01 MPa vacuum pressure, washed three times by autoclaved artificial seawater and resuspended with modified f/2 mediums containing limited (10 μmol L^−1^) or replete (882 μmol L^−1^) nitrogen (nitrate), and then inoculated in the dialysis bags (14 KD, 50 ml in volume). The dialysis bags were placed in a glass beaker containing 1 L of the same medium as in the dialysis bags. Then, the bags were incubated under three hydrodynamic conditions (0, 1.8 and 3.9 cm s^−1^) for 48 h. The diatom cell concentration was determined by a particle count and size analyzer (Z2, Beckman Coulter, United States), and its specific growth rate was calculated using the following equation:

$$\begin{array}{c} \mu \;\left(d^{-1}\right)=\;\frac{\ln N_{2}-\ln N_{1}}{t_{2}-t_{1}}, \end{array}$$ (6)

where N_1_ and N_2_ represent cell concentrations at *t*_*1*_ (initial) and *t*_*2*_ (after 2 days).

**FIGURE 2:**
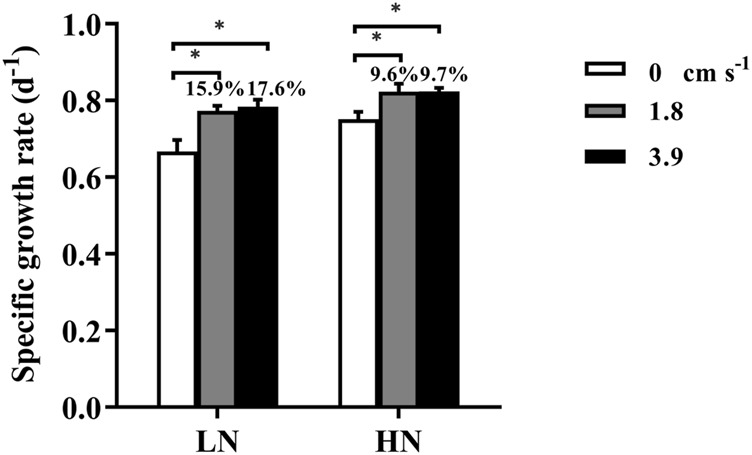
The specific growth rate (d^−1^) of the diatom *Thalassiosira weissflogii* grown within the dialysis bags (14 KD) under limited (10 μmol L^−1^, LN) and replete nitrate (882 μmol L^−1^, HN) and three water velocities (0, 1.8 and 3.9 cm s^−1^) determined over 48 h. Data are the means ± SD, *n* = 4 (4 independent dialysis bags). Values above the bars indicate increased proportions of the specific growth rate under water motion compared to that under motionless condition. One-way ANOVA and Tukey’s post-hoc test were employed to check the effects of water velocity on the specific growth rates. Asterisk indicates the significant differences at *p* < 0.05 level.

When there was no mixing in the incubation system, the specific growth rates (d^−1^) of *T.weissflogii* grown within dialysis bags under replete and limited nitrogen conditions were 0.75 ± 0.02 and 0.67 ± 0.03, respectively (Figure 2). Exposure to water motion significantly increased the specific growth rate of the diatom, since the water currents could reduce the thickness of the diffusion boundary layer and expedite the exchange of substances (Gao, 2021) (Figure 1). Consequently, the uptake and assimilation of nutrients and CO_2_ by the microalgal cells could be facilitated. The specific growth rate was significantly enhanced by nearly 18% under nitrogen-limitation but less than 10% under replete nitrate condition, indicating mixing could mitigate the inhibition of growth caused by nitrogen depletion. Although there was no significant difference in the growth rate between 1.8 and 3.9 cm s^−1^, the mean rates were higher with the faster current speed, especially under N-limited condition. Therefore, using dialysis bags to grow algae can take into account the effects of water motion or mixing on algal physiology.

### 2.4 Measuring Grazing Rates of a Heterotrophic Dinoflagellate by Applying the Dialysis Bags

**FIGURE 3:**
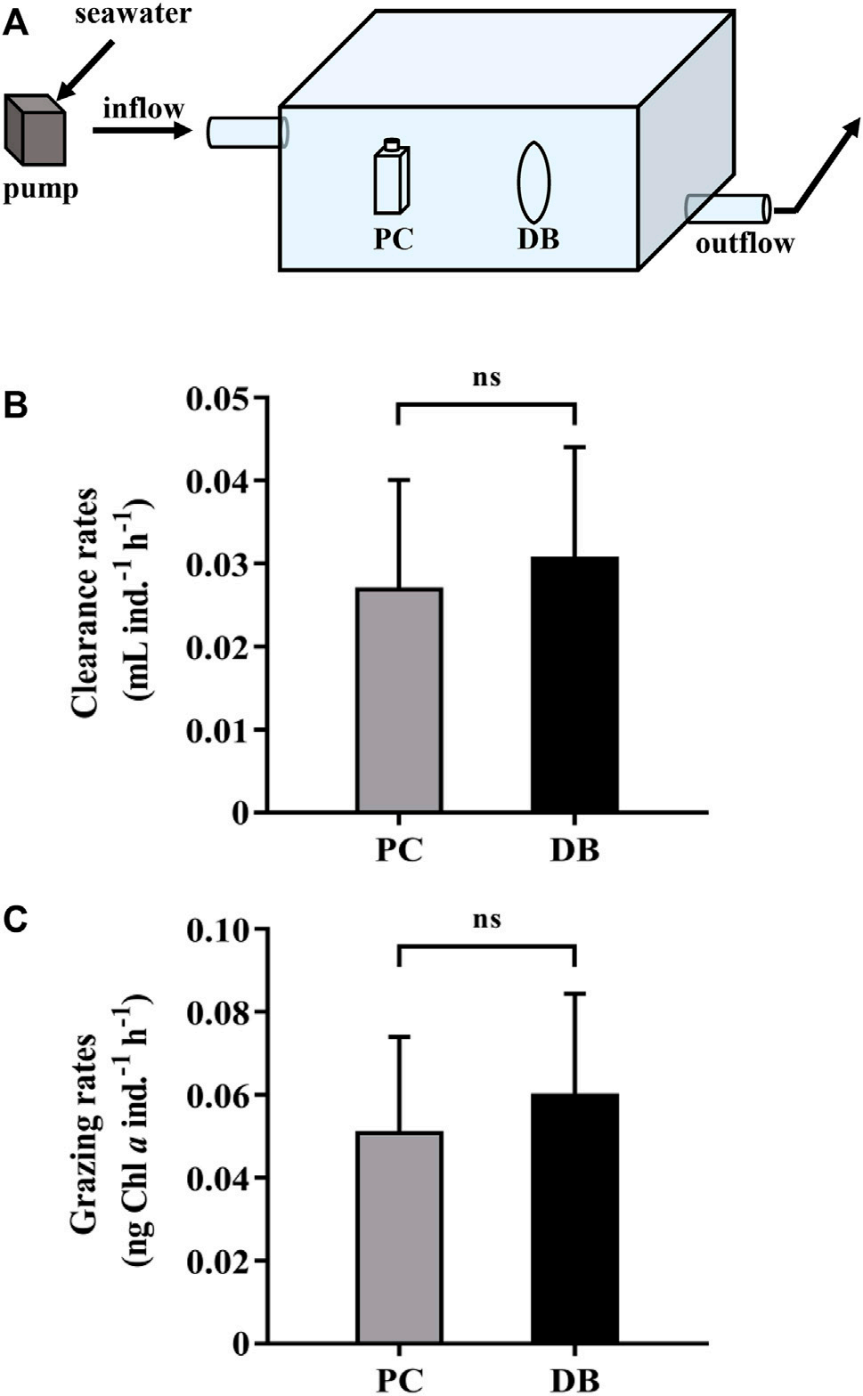
**(A)** A water bath with *in situ* seawater running through in which the dialysis bags and sealed bottles were incubated to measure and compare the grazing rates. **(B)** The clearance rates and **(C)** grazing rates of the heterotrophic dinoflagellates *Noctiluca scintillans* on natural phytoplankton assemblages during 1-day incubation by using polycarbonate bottles (PC, grey bars) and dialysis bags (DB, black bars). Data are the means ± SD, *n* = 3 (3 different replicates). Independent samples t-test was used to test the difference in the clearance and grazing rates of *N. scintillans* between the methods, and the differences were considered to be statistically significant at *p* < 0.05. The abbreviation ns stands for non-significant differences.

We carried out a field experiment to compare the grazing rates using dialysis bags and polycarbonate bottles. The heterotrophic dinoflagellate *Noctiluca scintillans* was isolated and collected in a subtropical coastal region, Wuyuan Bay, adjacent to the Facility for Ocean Acidification Impacts Study of Xiamen University (FOANIC-XMU) (24°31′48″N, 118°10′47″E), where the test was carried out. Subsequently, the cells of *N. scintillans* were transferred into 0.22 μm-filtered *in-situ* seawater for 24 h starvation treatment. On the day of the feeding experiment, 100 similarly sized *N. scintillans* cells were inoculated in the dialysis bags (14 KD, 600 ml) or PC bottles (600 ml) filled with 180 μm-filtered *in-situ* seawater with phytoplankton assemblages. The incubations were carried out simultaneously under incident solar radiation, and the bags or bottles free of *N. scintillans* were served as the controls. There were three replicates for each grazing test. Temperature was controlled similar to that of *in situ* by continuously running seawater through the water bath (Figure 3A). The water velocity in the bath was 2.2 cm s^−1^. After 24 h incubation, samples were equally taken from each container for chlorophyll *a* determination. The samples were filtered onto GF/F filters (Ф25 mm, Whatman, United Kingdom) and extracted in 3.5 ml acetone (90%, final concentration) at 4°C in darkness for 24 h. After centrifugation at 6,000 g for 10 min, the supernatants were measured using a Trilogy laboratory fluorometer (Turner Designs, United States) to determine Chl *a* concentration. Samples for measuring changed levels of nutrients were filtered through 0.45 μm cellulose acetate membranes, frozen immediately and stored at −20°C until later analysis. The concentration of dissolved inorganic nitrogen (DIN, nitrate plus nitrite), soluble reactive phosphorus (SRP) and silicate (Si(OH)_4_) were measured photometrically with a Technicon AA3 Auto-Analyzer (SEAL, Germany).The clearance and grazing rates of *N.scintillans* on natural phytoplankton were determined from the change in chlorophyll *a* concentration over time using the following equation according to Frost (1972):

$$\begin{array}{c} Clearance\;rates\;\left(mL\;{ind.}^{-1}\;h^{-1}\right)\;=\;\frac{V}{N}\;\times \;\frac{lnC_{t}^{\prime }\;-lnC_{t}}{\Delta t}, \end{array}$$ (7)

$$\begin{array}{c} Grazing\;rates\;\left(ng\;Chl\;a\;{ind.}^{-1}\;h^{-1}\right)\;=\frac{V}{N}\;\times \;\frac{lnC_{t\;}^{\prime }-lnC_{t}}{lnC_{t}\;–\;lnC_{0}}\;\times \;\frac{C_{t}\;–\;C_{0}}{\Delta t} \end{array},$$ (8)

where *V* is the volume of the containers, *N* is the number of grazers in each grazing group, Δt is the time interval of grazing, *C*_*0*_ is the initial Chl *a* concentration of phytoplankton, *C*_*t*_ and *C*_*t*_^′^ is the final Chl *a* concentration in grazing and control groups respectively.

The clearance and grazing rates of the heterotrophic dinoflagellate, *N.scintillans*, measured using the dialysis bags were respectively 0.031 ± 0.013 ml ind.^−1^ h^−1^ and 0.060 ± 0.024 ng Chl *a* ind.^−1^ h^−1^, slightly higher but not statistically different from those obtained by PC bottles, which were 0.027 ± 0.013 ml ind.^−1^ h^−1^ and 0.051 ± 0.023 ng Chl *a* ind.^−1^ h^−1^ (Figures 3B,C). At the end of the test, the nutrients in the dialysis bags maintained a relative high level, attributed to the replenishment by *in-situ* seawater, while DIN and SRP concentrations in the closed PC bottles were about 90% lower than those in the dialysis bags (Table 1). Consequently, using the dialysis bag to determine grazing rates is reliable even for prolonged incubation, since the membrane is permeable to nutrients and small molecules, changes of which could be tremendous in the sealed containers used in the traditional method (Table 1).

**TABLE 1:** The concentration of dissolved inorganic nitrogen (DIN, nitrate plus nitrite), soluble reactive phosphorus (SRP) and silicate [Si(OH)_4_] remained in the dialysis bags (DB) and polycarbonate bottles (PC) at the end of grazing experiment. The relative values in parenthese indicate decreased proportions of the nutrients in the bottles compared to that in the dialysis bags.

	DIN (μmol L^−1^)	SRP (μmol L^−1^)	Si(OH)_4_ (μmol L^−1^)
DB-control	1.168 ± 0.535	0.133 ± 0.046	4.826 ± 1.768
DB-grazing	1.496 ± 0.924	0.151 ± 0.065	5.019 ± 1.519
PC-control	0.072 ± 0.080 (94%)	0.010 ± 0.011 (92%)	3.985 ± 2.174 (17%)
PC-grazing	0.155 ± 0.108 (90%)	0.016 ± 0.005 (89%)	2.862 ± 0.325 (43%)

### 2.5 Measuring Grazing Rates of Copepods by Using Dialysis Bags in Laboratory

**FIGURE 4:**
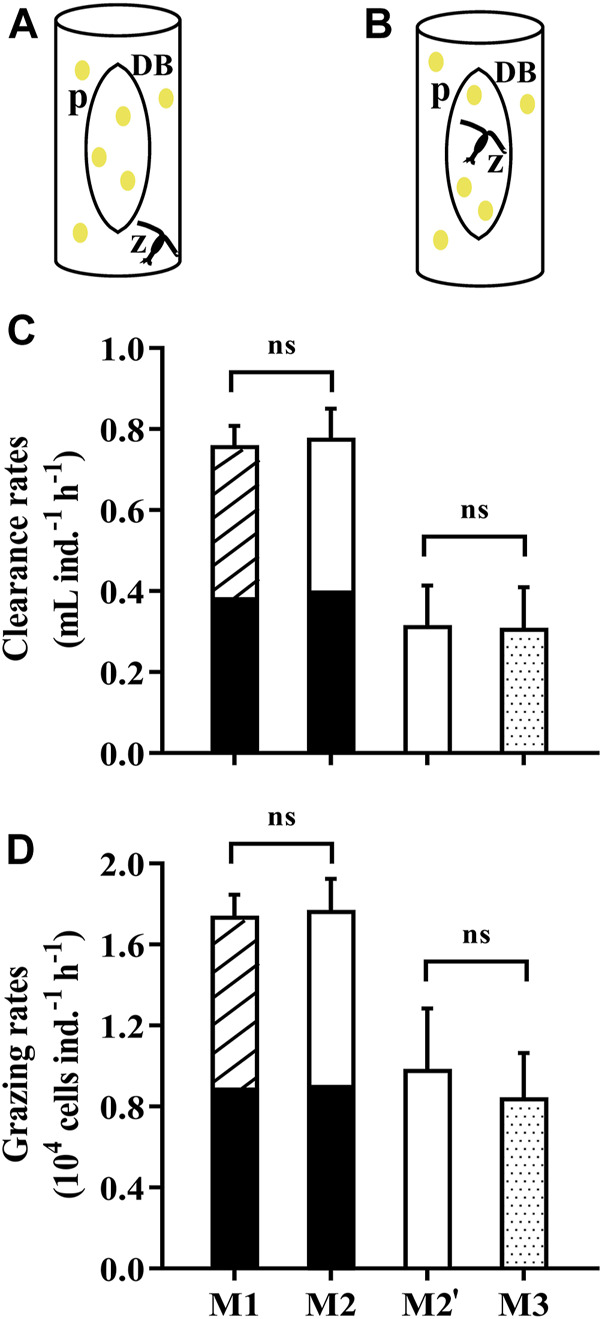
**(A)** The dialysis bags holding the microalga *Isochrysis galbana* as the control to determine the grazing rates of the copepod *Pseudodiaptomus* sp. in an open vessel (1L beaker). **(B)** The dialysis bags holding microalgal cells and the copepod to determine the grazing rates with the phytoplankton in the vessel as the control. The letter “p” represents microalgae, “z” zooplankton and “DB” dialysis bags. **(C)** The clearance rates and **(D)** grazing rates of the copepod *Pseudodiaptomus* sp. on the microalga *I.galbana* measured by using dialysis bags in different ways **(A,B)**. Specifically, M1 represents the traditional method using vessels with and without the prey, while M2 **(A)** and M3 **(B)** used dialysis bags within the vessels as the control or grazing tests, respectively. The comparison between M1 (striped over black bars) and M2 (white over black bars) was carried out over 8 h (4 h light, 4 h dark), while the comparison between M2’ (white bar) and M3 (dotted bars) was under the light conditions of 8 h. Data are the means ± SD, *n* = 3 (3 different replicates). Independent samples t-test was used to test the difference in the clearance rates and grazing rates between the methods, and significant differences were set at *p* < 0.05. The abbreviation ns stands for non-significant differences.

The traditional method (M1) for grazing experiments consists of three grazing vessels and one to three control vessels. Here, we designed a method by using a dialysis bag to separate the water in a beaker into two compartments, one for control (prey alone) and one for the grazing (grazer and prey). The microalgae grown within the dialysis bag is deemed as the control group while the zooplankton feed outside of the dialysis bag (M2, Figure 4A). In order to verify the feasibility and validity of this method (M2), we obtained the grazing rates of the copepod, *Pseudodiaptomus* sp., by M1 and M2. The copepods were collected from Longzhou pool, Xiamen, China and nursed under laboratory conditions. Prior to each grazing experiment, the mature individuals were selected and transferred into 0.22-μm filtered artificial seawater to be starved for 24 h. 120 copepods were aliquoted into 6 glass beakers (1 L) containing the microalgae *Isochrysis galbana* (initial concentration of 2.5×10^4^ cells mL^−1^). Simultaneously, *I. galbana* with the same concentration were introduced into one glass beaker (1 L) and three dialysis bags (60 ml), as the control group for M1 and M2 respectively. In parallel, we put the copepods inside the dialysis bags to graze and the microalgae residing outside of the dialysis bag were deemed as the controls (M3, Figure 4B). The test was conducted in the incubator for 8 h at 23°C either under 4 h light (40 μmol photons m^−2^ s^−1^) and 4 h dark (M1, M2) or 8 h light (M2', M3). It should be noted that only 10 copepods were placed into each dialysis bag in M3 due to its relatively small volume (60 ml). The clearance and grazing rates of copepods were determined by the equation in Section 2.4 while the parameter V (volume) equals to the space where the grazers feed.

During a period of 4 h light and 4 h dark, the clearance rates and grazing rates of *Pseudodiaptomus* were 0.78 ± 0.07 ml ind.^−1^ h^−1^ and 1.77 ± 0.15 × 10^4^ cells ind.^−1^ h^−1^ estimated by M2 and 0.76 ± 0.05 ml ind.^−1^ h^−1^ and 1.74 ± 0.10 × 10^4^ cells ind.^−1^ h^−1^ measured by M1 (Figures 4C,D), being comparable without significant difference. In contrast, after 8 h light incubations, the clearance rates and grazing rates of the copepods were respectively 0.31 ± 0.10 ml ind.^−1^ h^−1^ and 0.85 ± 0.22 × 10^4^ cells ind.^−1^ h^−1^ by using M3, and 0.32 ± 0.10 ml ind.^−1^ h^−1^ and 0.99 ± 0.30 × 10^4^ cells ind.^−1^ h^−1^ by M2′ (Figures 4C,D). The difference between the rates in these two apart comparison experiments (M1 and M2; M2′ and M3) may result from the collected copepods being at different life stages. The rates estimated by M3 were slightly but not significantly reduced from M2′. Since the ratio of inside/outside volume is 60/1,000, and the ratio of copepods number is 10/20, this dilution effect may cause different nutrients recycling regime and microalgae growth response. However, the difference between the rates measured by M2' and M3 was not detected, which may be ascribed to the relatively short experimental duration. Therefore, we suggest that using dialysis bags holding prey for control could obtain more reliable data.

### 2.6 Measuring Grazing Rates of Oysters in a Mesocosm Experiment

**FIGURE 5:**
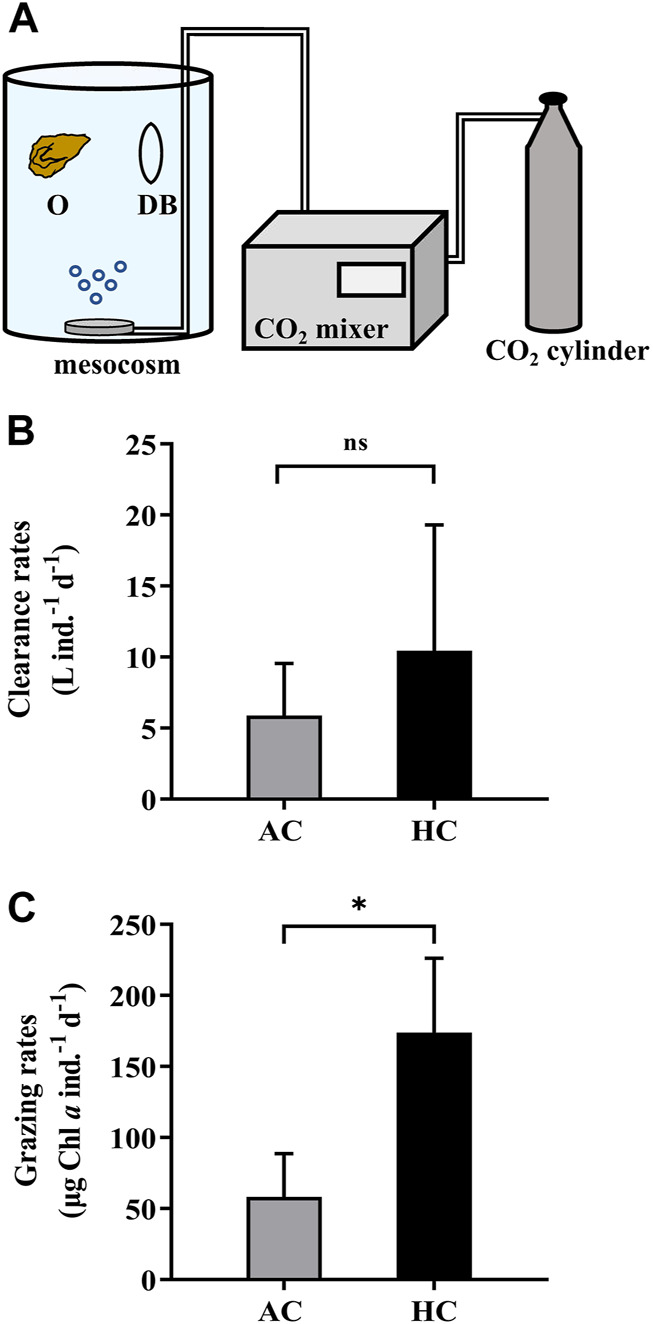
The dialysis bags with phytoplankton and zooplankton inside were employed in the mesocosms (3000 L) to determine the grazing rates by oysters **(A)**. The letter “O” represents oysters, and “DB” dialysis bags. The clearance rates **(B)** and grazing rates **(C)** of the oysters *Crassostrea angulata* on natural phytoplankton assemblages in the AC (ambient *p*CO_2_, 410 μatm, grey bars) and HC (high *p*CO_2_, 1,000 μatm, black bars) mesocosms. Data are the means ± SD, *n* = 4 (4 independent mesocosms). Independent samples t-test was used to test the difference in the clearance rates and grazing rates of *C. angulate* between *p*CO_2_ treatments. Asterisk indicates the significant difference at *p* < 0.05 level, and ns stands for non-significant differences.

We employed the second approach (M2) into a mesocosm study to verify its applicability in a more complex system. During 9 April 2021 to 21 May 2021, eight mesocosms (Figure 5A) were set up on the platform of FOANIC-XMU. Each transparent thermoplastic polyurethane (TPU) bags were filled with 3000 L 0.01 μm filtered *in-situ* surface seawater by a water purifier (MU801-4T, Midea, China). Four of the eight mesocosms were set as the control groups with ambient level of *p*CO_2_ (AC, 410 μatm), while the other four mesocosms were set as the elevated *p*CO_2_ (HC, 1,000 μatm) treatment groups by using a CO_2_ mixer (CE-100B, Wuhan Ruihua, China) (Huang et al., 2021). Subsequently, 100 L 180 μm filtered *in-situ* seawater was simultaneously added into each mesocosm to construct a natural microflora. After 16 days, 36 *Crassostrea angulate* juveniles (3–5 months old, shell length = 34.13 ± 4.89 mm, shell height = 19.96 ± 3.77 mm) were evenly placed into three net cages and hanged in each mesocosm in the presence of other grazers.

To conduct the grazing experiment for *C. angulate*, two dialysis bags were placed and fixed in each mesocosm. Specifically, 2 L sample collected from each mesocosm bag was mixed adequately, and then a portion of it was filtered onto GF/F filters (Ф25 mm, Whatman, United Kingdom) for determination of chlorophyll *a* concentration before grazing (C_0_), while another portion were injected into each dialysis bag (600 ml) as the controls in the absence of oyster. Since the changes of chlorophyll *a* concentration in mesocosms and the dialysis bags were attributed to the growth of phytoplankton and the predation of zooplankton and oysters, samples taken from each mesocosm and each of the dialysis bags in 24 h were filtered for measuring the chlorophyll *a* concentrations. The chlorophyll *a* concentration was determined according Ritchie (2006). The clearance and grazing rates of the oysters were calculated by the equations in Section 2.4, except that the parameter V (volume) represents the volume of the mesocosm.

It was found that the elevated *p*CO_2_ induced a significantly higher grazing rate of oysters (173.82 ± 52.37 μg Chl *a* ind.^−1^ d^−1^), which was about three times that under ambient *p*CO_2_ level (58.31 ± 30.43 μg Chl *a* ind.^−1^ d^−1^) (Figures 5B,C).

### 2.7 Statistical Analysis

Statistical analyses were performed by SPSS 26.0 software. The homogeneity of variance was examined using Levene’s test before the statistical analyses. One-way ANOVA and independent samples t-test were employed to establish differences among treatments. Difference was considered to be statistically significant at *p* < 0.05.

## 3 Conclusion and Recommendations

During the incubations based on the traditional prey biomass subtraction method (Frost, 1972), levels of nutrients, pH, *p*CO2 and *p*O2 as well as other chemicals can be altered due to biological processes within the closed water bodies. Using the dialysis bags can improve such bottle effects since the membrane allows exchanges of gases and chemicals. On the other hand, the effects of ultraviolet irradiances on the marine primary and secondary producers have been neglected (Gao et al., 2019), since incubation bottles used for biological investigations are opaque to solar UV radiation. The dialysis membrane allows the transmission of both photosynthetically active radiation (PAR) and UV radiation (Supplementary Figure S1). Therefore, the dialysis bags can be applied in examining the impacts of ocean acidification, hypoxia, and UV radiation on aquatic plankton, since they can easily be placed under exposures to these factors. From the economic perspective, using dialysis bags to measure the grazing rates of secondary producers could reduce the cost by minimizing the number of containers or replicates required for the traditional method. We conclude that using dialysis bags to grow microalgae and to determine grazing rates has the following merits: 1) Reducing the bottle effects due to allowed exchanges of chemicals; 2) enabling comparisons of grazing rates among different grazers within the same environment (mesocosm, microcosm); 3) taking water current speed into account, since mixing or water velocities affect biological activities; 4) being suitable to examine growth rates of microalgae and secondary producers’ grazing activities under different levels of CO_2_ and O_2_ while maintaining stable gas partial pressures within and outside of the dialysis bags.

In application of dialysis bags, the following recommendations should be considered:

1. In Section 2.2, we provided a simple model to determine the diffusion property of the membrane. Based on the data (Figure 1), measuring the changes in solute concentration within 3–5 h at 3 time points is enough to derive a reliable apparent diffusion coefficient. The membranes with a higher apparent diffusion coefficient result in higher exchange rates of nutrients and small molecules. Therefore, one can choose proper dialysis membranes for different exchanging rates to examine different biological or biogeochemical processes.
2. Dialysis membrane should be cleaned thoroughly to prevent the experiment system from contamination.
3. Since the epiphytic microorganisms and sticky colloidal particles may adhere to the surface of the membrane due to biofouling, exchanging rates of gases or nutrients could be hindered. Therefore, replacement with new dialysis bags periodically is recommended, if the incubation lasts too long. Inversely, the dialysis bags could be applied to test biofouling effects on biological activities of organisms or communities.
4. The dialysis bags can be used in combination with a bubbled zooplankton grazing rotating wheel (Kim and Kang, 2003) or other agitation apparatus to enhance internal water mixing, facilitating the exchange of nutrients, gases, and metabolites. Therefore, this approach with the dialysis bags can be used to study the effects of water current speed or mixing rates in dynamic water conditions.
5. Since the dialysis bags can be used to measure grazing rates of several different grazers or different groups of secondary producers at the same time in the same environment (Figure 5A), this approach can be expanded to explore grazing rates of different trophic levels simply by combining prey and grazer (or grazers) in the same testing and control dialysis bags. The grazing rates by different grazers or different groups of grazers can be estimated with the same calculation as prey biomass subtraction method (Frost, 1972). To do this, all the dialysis bags should be maintained in the same chemical and physical environments.
6. Before taking subsamples, the dialysis bags should be inverted repeatedly to obtain a homogenized internal cell or individual biomass density.
7. There may be needs to perform the grazing tests using the dialysis bags of larger “pore size,” inquiries can be directed to the membrane manufacturers.
8. Since the dialysis bags are commercially available at very low price, pre-test for applying this method is recommended for different kinds of experiments.

## Data Availability

The raw data supporting the conclusions of this article will be made available by the authors, without undue reservation.
