# Corrigendum: Applying Dialysis Bags to Grow Microalgae and Measure Grazing Rates by Secondary Producers

**DOI:** 10.3389/fphys.2022.940603

**Published:** 2022-06-15

**Authors:** Yang Tian, Xiangqi Yi, Kunshan Gao

**Affiliations:** ^1^ State Key Laboratory of Marine Environmental Science, College of Ocean and Earth Sciences, Xiamen University, Xiamen, China; ^2^ Co-Innovation Center of Jiangsu Marine Bio-Industry Technology, Jiangsu Ocean University, Lianyungang, China

**Keywords:** grazing rate, phytoplankton, zooplankton, secondary producer, dialysis bag

In the original article, there was an error in the second paragraph of **Section 2.4: Measuring Grazing Rates of a Heterotrophic Dinoflagellate by Applying the Dialysis Bags**. The error occurred in the unit of grazing rates of *Noctiluca scintillans*, **μ**g Chl *a* ind.^−1^ h^−1^, which should be corrected as **n**g Chl *a* ind.^−1^ h^−1^.

The corrected paragraph is as follows:

“The clearance and grazing rates of the heterotrophic dinoflagellate, *N. scintillans*, measured using the dialysis bags were respectively 0.031 ± 0.013 mL ind.^-1^ h^-1^ and 0.060 ± 0.024 ng Chl *a* ind.^-1^ h^-1^, slightly higher but not statistically different from those obtained by PC bottles, which were 0.027 ± 0.013 mL ind.^-1^ h^-1^ and 0.051 ± 0.023 ng Chl *a* ind.^-1^ h^-1^ (Figure 3B, C). At the end of the test, the nutrients in the dialysis bags maintained a relative high level, attributed to the replenishment by *in-situ* seawater, while DIN and SRP concentrations in the closed PC bottles were about 90% lower than those in the dialysis bags (Table 1). Consequently, using the dialysis bag to determine grazing rates is reliable even for prolonged incubation, since the membrane is permeable to nutrients and small molecules, changes of which could be tremendous in the sealed containers used in the traditional method (Table 1).”

In there was the same error in Figure 3C as published. The unit of grazing rates of *Noctiluca scintillans*, **μ**g Chl *a* ind.^−1^ h^−1^, should be corrected as **n**g Chl *a* ind.^−1^ h^−1^. The corrected Figure 3 appears below.

**FIGURE 3:**
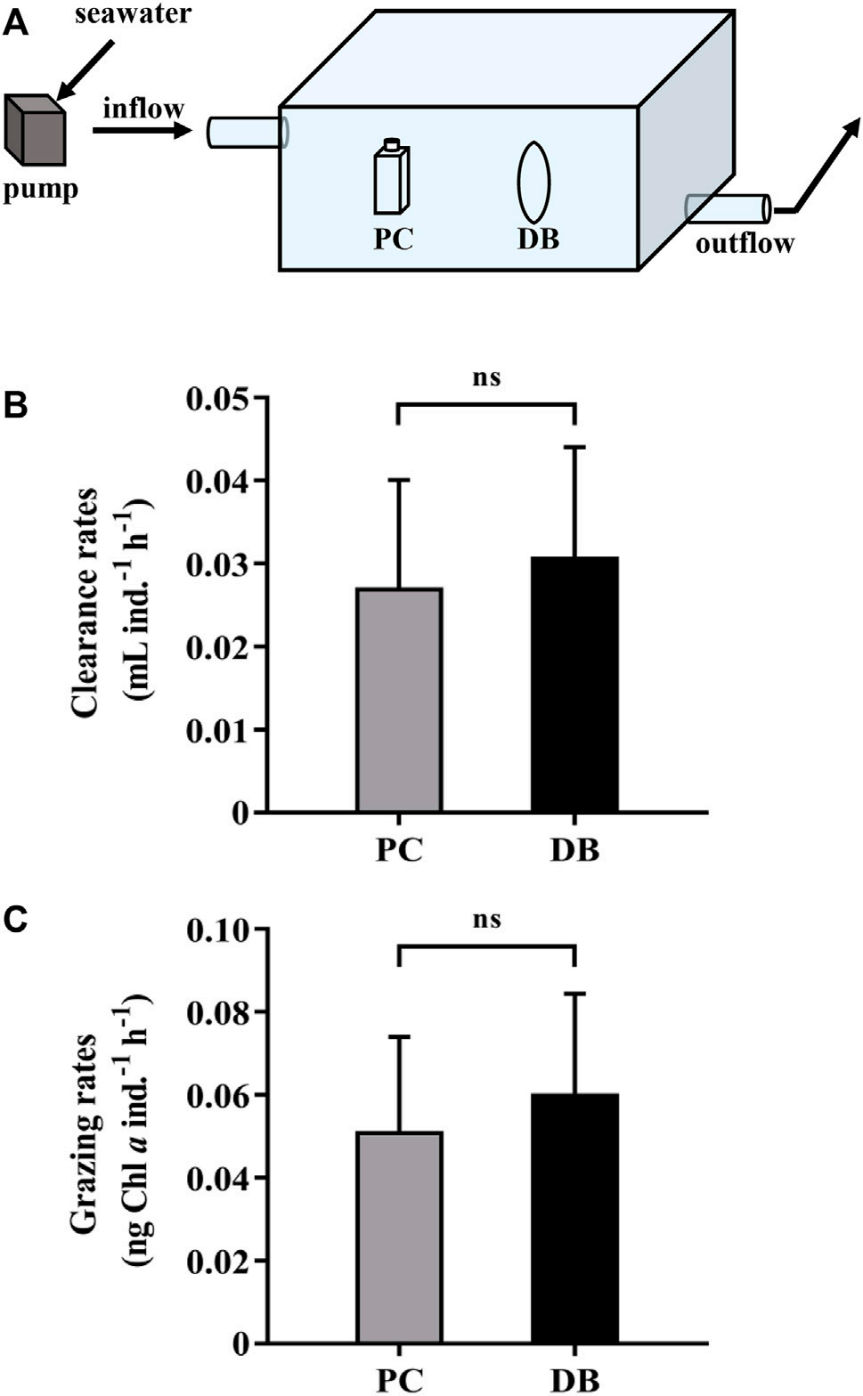
**(A)** A water bath with *in situ* seawater running through in which the dialysis bags and sealed bottles were incubated to measure and compare the grazing rates. **(B)** The clearance rates and **(C)** grazing rates of the heterotrophic dinoflagellates *Noctiluca scintillans* on natural phytoplankton assemblages during 1-day incubation by using polycarbonate bottles (PC, grey bars) and dialysis bags (DB, black bars). Data are the means ± SD, *n* = 3 (3 different replicates). Independent samples *t*-test was used to test the difference in the clearance and grazing rates of *N. scintillans* between the methods, and the differences were considered to be statistically significant at *p* < 0.05. The abbreviation ns stands for non-significant differences.

The authors apologize for this error and state that this does not change the scientific conclusions of the article in any way. The original article has been updated.

