# Supplementary material for: Applying Dialysis Bags to Grow Microalgae and Measure Grazing Rates by Secondary Producers
Source: Front Physiol. 2022 May 10;13:838001. doi: 10.3389/fphys.2022.838001 (PMC9127382; doi:10.3389/fphys.2022.838001)
Supplement: Supplementary file 1 [file Table1.DOCX]

Supplementary Material

# Supplementary Figures

**
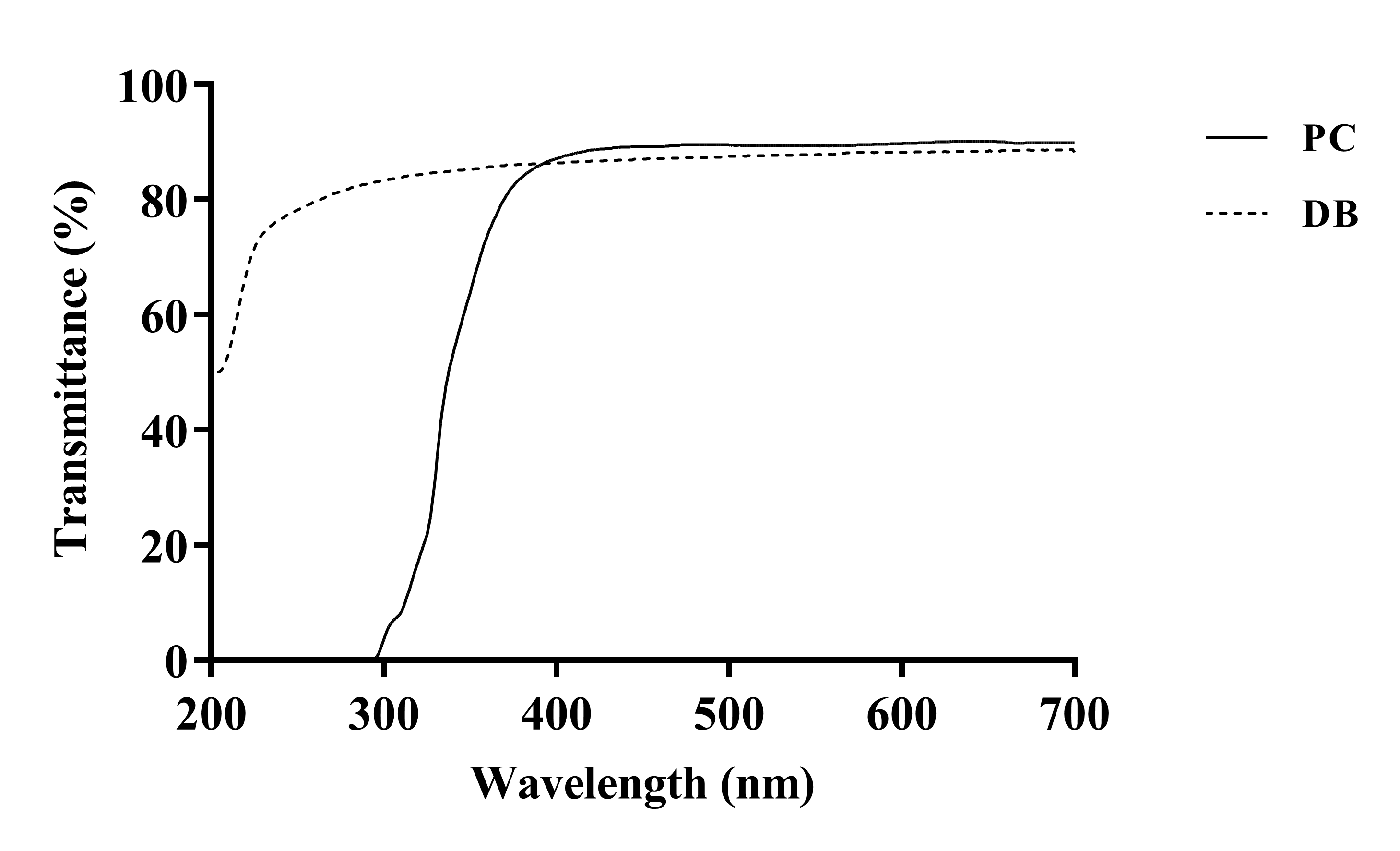
**

**Supplementary Figure 1.** The transmission spectra of dialysis bags (DB, dotted line) and polycarbonate bottles (PC, solid line) used in the experiment.
